# Patient engagement in the development and delivery of healthcare services: a systematic scoping review

**DOI:** 10.1136/bmjoq-2023-002309

**Published:** 2023-06-27

**Authors:** Joachim Støren Sagen, Geir Smedslund, Ann Elisabeth Simonsen, Andreas Habberstad, Ingvild Kjeken, Hanne Dagfinrud, Rikke Helene Moe

**Affiliations:** 1 NKRR, Center for treatment of Rheumatic and Musculoskeletal Diseases (REMEDY), Diakonhjemmet Hospital, Oslo, Norway; 2 Oslo Metropolitan University, Faculty of Health Sciences, Oslo, Norway; 3 Norwegian Institute of Public Health, Oslo, Norway; 4 Røysumtunet Rehabilitation Centre, Oslo, Norway; 5 The Norwegian Federation of Organisations of Disabled People, Oslo, Norway; 6 University of Oslo, Faculty of Medicine, Oslo, Norway

**Keywords:** Patient Participation, Health Equity, Healthcare quality improvement, Shared decision making

## Abstract

**Background:**

Patient engagement (PE) is required to improve future healthcare services. PE in the development and delivery of healthcare services is likely to be complex but is scarcely described.

**Objectives:**

The objective of this scoping review was to summarise primary studies on mesolevel PE regarding structure, process and outcomes. More specifically, the aim was to explore barriers and facilitators to successful PE, how persons are engaged in the process and summarise reported consequences.

**Method:**

A systematic scoping review was conducted, searching the MEDLINE, EMBASE, Cochrane and PsycINFO databases. Primary studies, published between 7 July 2005 and 4 October 2022, were considered for inclusion. Two reviewers extracted data about PE (eg, attributes of PE settings, facilitators and barriers, and outcomes to PE) and the first author coded the extracted data into structural, processual and outcome themes.

**Results:**

Of 8588 identified records, 37 studies were eligible. Most of the included studies were conducted in Europe (n=19; 51%) and North America (n=13; 35%). Structures that ensure sufficient stakeholder representativeness and PE knowledge through education may facilitate the PE process further, regardless of the environmental setting. Interpersonal relationships with uneven power dynamics were reported as noteworthy processual barriers to meaningful PE, while clearly described roles and tasks were reported as important facilitators. In contrast to hard outcomes with operationalised PE effects, the most noteworthy outcomes of PE were reported as soft processual consequences such as patient representatives improving their self-esteem and feeling valued.

**Conclusions:**

Unfortunately, there is a dearth of studies exploring hard and operationalised PE outcomes on healthcare services and patients receiving healthcare. The PE process may be facilitated by dedicated finances to PE education and by ensuring sufficient stakeholder representativeness.

**WHAT IS ALREADY KNOWN ON THIS TOPIC**

- The existing literature suggests possible procedural mechanisms and structural attributes for evaluating the quality of mesolevel patient engagement (PE) and its consequences. This study is needed to provide knowledge on current PE and the influence of procedural mechanisms present in current healthcare structures, including approaches, practices and outcomes.

**WHAT THIS STUDY ADDS**

- Sufficient finances, earmarked PE education and training, and stakeholder representativeness are of great structural importance to facilitate meaningful PE approaches, which in turn may improve all stakeholders’ processual experiences.

**HOW THIS STUDY MIGHT AFFECT RESEARCH, PRACTICE OR POLICY**

- The findings from this review shed light on structural attributes that hinder and facilitate meaningful PE processes and may serve as a basis for the development of educational initiatives targeting mesolevel PE to ease the distribution of future PE responsibilities. The results may also help guide future research aiming to test the rigour of outcome measures used to evaluate and compare PE initiatives in, and across, healthcare services.

## Introduction

### Rationale

The implementation and evaluation of patient engagement (PE) includes a number of concepts and dimensions with multiple possible terms and definitions.^1^ In this review, the term (PE) will be used when referring to comanagement of healthcare services. PE can be understood as patients, patient representatives or patient organisations engaging with stakeholders at different levels of care.^2^ PE is highlighted as an important part of healthcare services and as a criterion for quality.^3 4^

**Figure 1:**
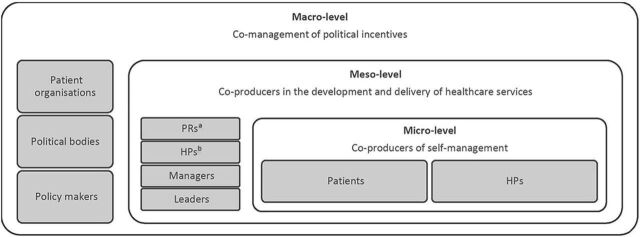
A brief overview of PE stakeholders at the microlevels, mesolevels and macrolevels in healthcare services, inspired by Andreassen.^2^ ^a^PRs, patient representatives, including carers. ^b^HPs, healthcare professionals.

PE can take place at the microlevel, mesolevel and the macrolevel. At the microlevel, patients are coproducers of self-management, while the comanagement of political incentives on a governmental level can be referred to as the macrolevel. At the mesolevel, patient representatives engage as coproducers in the development and delivery of healthcare services, aiming to improve these services for a larger group of individuals.^2^ As illustrated in figure 1, PE is suggested to involve one or more of the professionals working in healthcare services in addition to patient representatives such as patients and carers.^1^

Historically, PE initiatives have focused on patients as coproducers of their own care. Over the last decades, there has been increasing attention on PE in the design, implementation and delivery of healthcare services at the mesolevel. PE at this level is often organised as collaborative working groups, patient councils or patient advisory boards. The outcomes of PE are likely to be complex and may, in different ways, influence both the healthcare service and the persons involved.^5 6^ PE has the potential to improve shared decision-making, person-centred care and organisations’ communication.^7–9^ It has also been argued that PE may decrease hospital admissions and reduce costs if performed as a meaningful cocreation process.^10^

To inform future research and practice, a scoping review was performed to systematically identify and map existing gaps in knowledge regarding the structure, process and outcome of PE. We used a PE-adapted version of Donabedian’s model to evaluate the quality of healthcare services to increase the understanding of current worldwide PE practices.^11^ This review will focus on PE structures (the characteristics of settings or contexts in which PE occurs), PE processes (what PE practices and mechanisms are actually performed) and outcomes (the consequences on healthcare services, stakeholder relationship and experiences as a consequence of PE practices). PE regarding peer support initiatives, research and records focusing on patient or staff education as single PE practices will not be considered.

### Objectives

The objective of this scoping review was to identify, summarise and map primary studies on mesolevel PE regarding structure, process and outcomes, following the Preferred Reporting Items for Systematic Reviews and Meta-Analyses (PRISMA) checklist for scoping reviews.^12^

The scoping process was guided by the following research questions:

- How are different stakeholders engaged in the process?
- What hinders and facilitates successful PE?
- What are the reported PE outcomes?

## Methods

A systematic scoping review was conducted based on the framework by Arksey and O’Malley and Levac *et al*,^13 14^ following the required five stages: (1) identifying the research question, (2) identifying relevant studies, (3) study selection, (4) charting the data and (5) collating, summarising and reporting the results.

### Patient and public involvement

One patient representative (AH) affiliated with the Norwegian Federation of Organisations of Disabled People (FFO) and one representing the patient advisory board at Røysumtunet Rehabilitation Centre (AES) were actively involved as patient research partners. They were engaged from the very beginning and were dedicated to the importance of the research topic during all stages of the research stages. Moreover, they participated in developing the project plan and study design, helped interpret the findings, prepare the manuscript, and will contribute to the dissemination of results to patient organisations and policy-makers.

### Protocol and registration

The protocol was drafted using the PRISMA-Protocols^15^ applicable to the scoping review methodology. The protocol was registered prospectively in the Open Science Network on 8 October 2020 (10.31219/osf.io/ysa9v) and revised by the research team due to increased familiarity with the research area.

### Identifying the research question

Because of the broadly defined research purpose, we identified key elements by using frameworks such as (but not limited to) Population, Context and Concept.^16^ Population criteria included patient representatives and healthcare providers. Context criteria comprised practices and motives, whereas concept criteria were PE in the development and delivery of healthcare services.

### Identification of studies

#### Eligibility criteria

Primary studies published between 7 July 2005 and 4 October 2022 that matched the inclusion criteria were considered. Studies in English, Norwegian, Swedish or Danish were eligible. The studies could indirectly or directly involve patients, including carers, as well as other representatives from the health service (eg, healthcare professionals, managers, leaders). Studies were included if participants were 16 years or older with adequate consent competence.

After a preliminary search performed in 2019, databases were prioritised through team discussion. The search strategies were refined through team discussion and drafted by an experienced medical librarian. A comprehensive search was performed from 1 January 2005 to 6 July 2020 in the following bibliographic databases: MEDLINE, EMBASE, Cochrane and PsycINFO to identify potentially relevant primary studies. To manage our findings according to available resources and to explore the latest developments in PE, our primary focus was on publications from the previous fifteen years. A new search was performed for 1 June 2020 to 4 October 2022 to ensure the inclusion of the newest published studies. The search strategy for the last search is attached as online supplemental file 1.

10.1136/bmjoq-2023-002309.supp1 Supplementary data

### Selection of studies

After duplicates were removed, the remaining records underwent title and abstract screening by two researchers (GS and JSS), and in cases of disagreement, a third reviewer was consulted (RHM). When inclusion or exclusion could not be determined based on the title and abstract, the article was screened in full text. Three reviewers performed the full text screening (GS, RHM and JSS).

**Figure 2:**
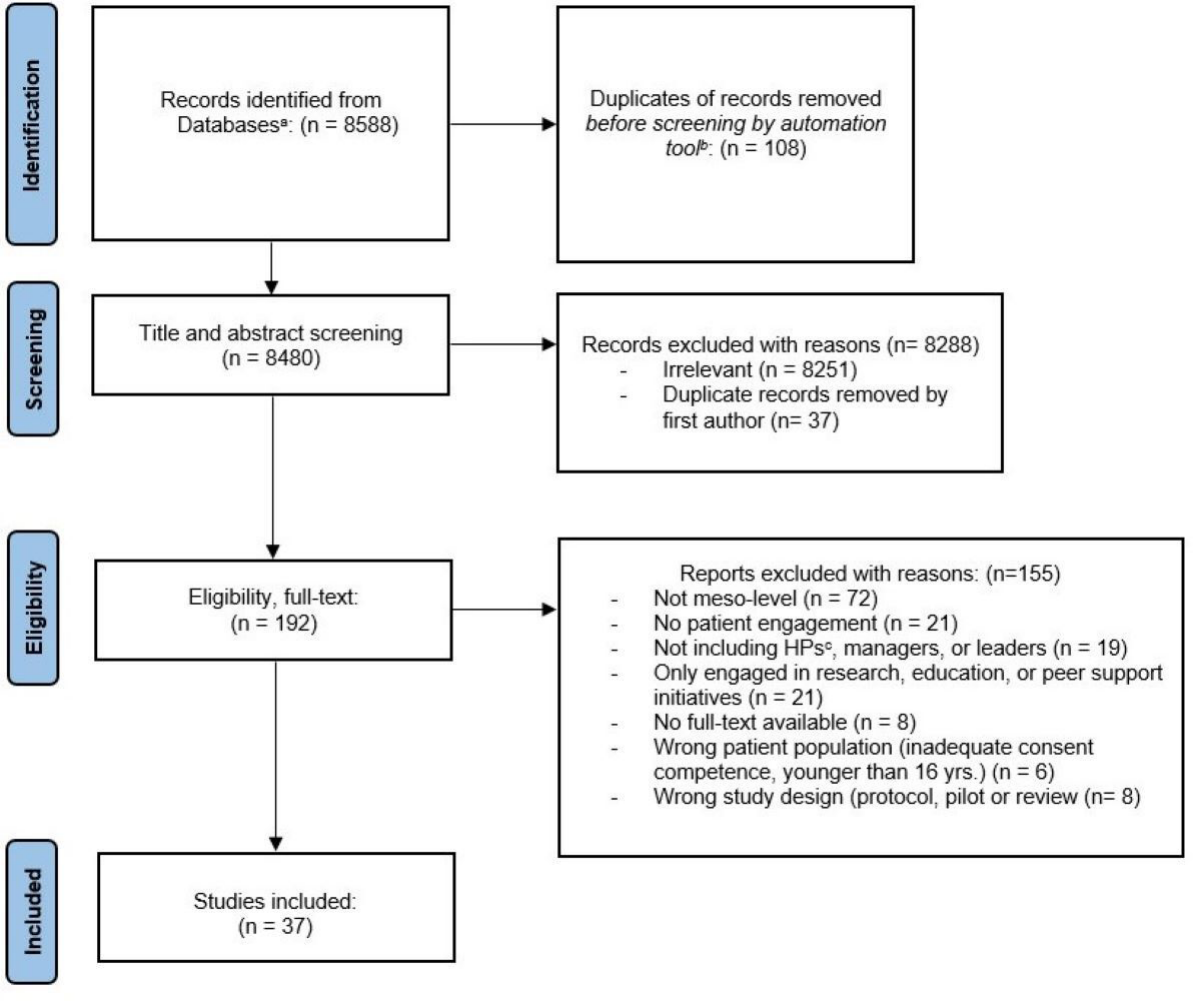
Flow chart for identification of studies via databases. ^a^MEDLINE, EMBASE, Cochrane and PsycINFO. ^b^Covidence. ^c^HPs, healthcare professionals.

### Data

A data-charting form was developed and adjusted to the included studies by two reviewers using Covidence^17^ (GS and JSS). When a primary study was found eligible, data were extracted independently by two authors (GS and JSS). In case of conflicts, the first author (JSS) made the final decision. The reviewers extracted data about PE characteristics and mechanisms (eg, facilitators, and barriers to PE, including structural attributes, and stakeholder behaviours and experiences). The environmental setting (health and longevity, citizens’ knowledge and standard of living) of the study’s country of origin were compared using reports from the Human Development Index (HDI),^18^ which measures key dimensions of human development.

The first author (JSS) coded the extracted PE characteristics into themes using Quirkos V.2.4.1.^19^ The themes were guided by the proposed framework^11^ for evaluating PE initiatives at the mesolevel. Included studies were quality assessed by two independent reviewers in pairs (RHM, GS and JSS) using the nine first questions of Critical Appraisal Skills Programme,^20 21^ which is suitable for randomised controlled studies^20^ and qualitative studies.^21^ The Mixed Methods Appraisal Tool^22^ was used to assess quality for mixed method studies, cross-sectional studies and case reports, and disagreement was solved by group discussion. The response categories C=Can’t tell and N=No were collapsed to No. Based on the number of yeses and noes for each primary study, an overall assessment was performed (presented in table 1). Robvis^23^ was used to create visual quality assessment tables as shown in online supplemental file 2.

10.1136/bmjoq-2023-002309.supp2 Supplementary data

**Table 1:** Characteristics of the included studies

Study, ref	Country	Method/design (intervention/ comparator)	Study PE aim	Participants (N=2992)	Quality*
			***Experiences, attitudes or opinions***		
Amann *et al* 2018^24^	Switzerland	Qualitative	How HP/management experience PE.	HP/management (22)	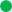
Brouwers *et al* 2017^25^	Canada	Mixed	Attitudes towards practice guideline development.	PR (41)	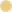
Gagliardi *et al* 2008^26^	Canada	Qualitative	Indicator selection.	PR (15), HP (10), Management (5)	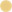
Gurung *et al* 2017^27^	Nepal	Qualitative	System strengthening.	PR (24)	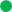
Lindblom *et al* 2021^28^	Sweden	Qualitative	Codesign process	PR (4), HP (10)	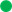
Livingston *et al* 2013^29^	Canada	Mixed	Improve care.	PR (25), HP (27)	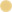
Neech *et al* 2018^30^	UK	Qualitative	Experiences of involvement.	PR (13)	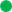
Rise *et al* 2014^51^	Norway	Qualitative	Implementation of a comprehensive development plan.	PR (4), HP, managers/leaders (13)	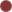
Samudre *et al* 2016^56^	India	Qualitative	Experiences, barriers and facilitators.	PR (8), HP (3) managers/leaders (16)	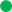
			***Development of recommendations***		
Armstrong *et al* 2017^31^	USA	Qualitative	Guideline development.	PR (15)	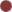
Armstrong *et al* 2018^32^	USA	RCT (patients and physicians/physicians)	Guideline question formation.	PR, HP (19)	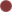
Fraenkel *et al*, 2016^60^	USA	Case report	Develop clinical practice guideline recommendations	PR, HP (10)	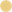
Goodman *et al* 2017^47^	USA	Qualitative	Clinical practice guideline development.	PR (11)	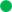
Boivin *et al* 2014^52^	Canada	RCT (priority setting with PE/not PE)	Assess the impact.	PR (83), HP (58), management (31)	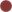
			**Impact**		
de Souza *et al* 2017^53^	UK	Qualitative/case report	Development of innovative strategies	PR (10)	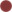
Daouk-Öyry *et al* 2018^54^	Lebanon	Qualitative/action research	Engaging the patient in cocreating.	PR (18)	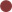
Dickinson *et al* 2020^57^	USA	RCT (standard/plusPE)	Adoption of evidence-based guidelines	Other (Practices) (211)	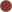
O'Donnell *et al* 2019^33^	Ireland	Qualitative	Quality improvement initiatives.	PR (10), HP (8)	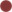
Greene *et al* 2018^34^	USA	Mixed	Influence on quality improvement.	Focus group/survey: PR (17)/(47), leaders (11)/(56)	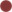
Gremyr *et al* 2018^59^	Sweden	Quantitative/Cross-sectional	Radicality of improvement	HP (155)	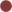
Omeni *et al* 2014^35^	UK	Mixed	Views on impact.	PR (302), HP (143)	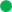
Scholtes *et al* 2021^36^	UK	Quantitative/cross-sectional	Occurrence and influence	HP (35), management (29)	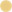
			***Exploring, understanding and knowledge***		
Anderson *et al* 2021^37^	Canada	Qualitative	Approaches and strategies	PR (20) HP (10), managers/leaders (10)	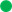
Carlsson *et al* 2007^48^	Sweden	Qualitative	Generate knowledge about quality improvement.	PR (16), HP (10)	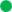
Dayekh *et al* 2022^38^	Lebanon	Qualitative	Benefits and barriers	PR (41), HP (27)	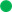
Fudge *et al* 2008^39^	UK	Qualitative	Policy.	PR (158), HP (18)	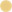
Galvin *et al* 2020^40^	UK	Qualitative Action research	Insights and benefits	PR (12), HP (17)	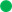
Hashem *et al* 2018^49^	UK	Qualitative	Decisions about funding medicines.	PR, HP, other (41)	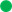
Hwang and Warshaw, 2019^41^	USA	Cross-sectional	Clinical settings.	HP (829)	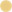
McKevitt *et al* 2018^42^	UK	Qualitative	PE in major system changes.	PR, HP, managers/leaders, other (45)	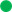
Rise *et al* 2013^50^	Norway	Qualitative	Service users’ and service providers’ own definitions.	PR (4) HP (33), management (44)	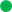
Sharma *et al* 2018^43^	USA	Qualitative	Define roles and understand recruitment.	Managers/leaders (19)	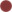
Steffensen *et al* 2022^58^	Denmark	Qualitative	Practices and influence	PR (3), HP (1), management (8)	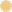
van der Meide *et al* 2015^44^	The Nederlands	Case report	The client council.	PR (9)	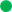
Weiste *et al* 2021^45^	Finland	Qualitative	Workshop contributions	PR (9), HP (38), management (7),	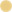
Whiston *et al* 2019^46^	Ireland	Qualitative	Intensity and implementation	PR (22), HP (9)	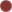
Woelders and Abma 2019^55^	The Nederlands	Qualitative action researrch	Alternatives to formal involvement	PR (10), management (1), HP (2)	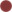

*Judgement: 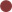 Poor quality 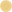 Fair quality 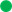 Good quality. The response categories C=Can’t tell and N=No used for the Critical Appraisal Skills Programme and the Mixed Methods Appraisal Tool were collapsed to No, <2 ‘no’=good quality, <3 ‘no’=fair quality and ≤3 ‘no’= poor quality.

HP, health professionals; PE, patient engagement; PR, patient representatives; RCT, randomised controlled trial.

## Results

### Selection of sources of evidence

Initial searches detected 8588 records about PE at the mesolevel, of which 145 were duplicates. After title and abstract screening, 192 records were assessed for full text review, of which 37 studies met the eligibility criteria. Most of the excluded records (n=72) did not address the mesolevel and forty records did not involve comanagement between patient representatives, healthcare professionals, managers or leaders. Twenty-one records were excluded due to out-of-range settings (eg, research, peer support or education as single PE initiatives). Reasons for excluding records were no full text available (n=8), wrong study design (n=8) (protocol, review or pilot), or inaccurate patient population (n=6) (inadequate consent competence, target population younger than 16 years) (figure 2).

### Sources of evidence

Of the 37 included studies, 33 (89%) were conducted in countries that scored ‘very high’ on the HDI over the last decade. As shown in table 1, 24 (65%) studies were qualitative; of these, 3 used an action research design (8%) and 2 (5%) used a case-study design. Four (11%) studies used mixed methods, three (8%) studies were randomised controlled trials, three (8%) were cross-sectional studies and one (3%) was a case report. Most studies (n=34;92%) were published within the last 10 years, with the largest share deriving from Europe (n=19;51%) and 13 (35%) originating from North America. The countries with the most included studies were the UK (n=8;22%) and the USA (n=8;22%). Two (5%) studies were developed in Lebanon, one (3%) in India, and another in Nepal (3%). The Quality appraisal detected 15 good quality, 9 fairly good and 12 poor quality studies. A summary of grading of the quality of evidence is included in online supplemental file 2.

The aims of the included studies were to explore PE at the mesolevel to increase knowledge and understanding about how PE was performed (n=15;41%), the impact of PE initiatives (n=8;22%), or experiences, attitudes or opinions of PE (n=9;24%). All studies aiming to assess the outcome of involving patient representatives in the development of treatment recommendations originated from North America (n=5;14%). The aim in most studies was to increase knowledge about PE activities in the field of mental healthcare (n=8;20%). Three studies (8%) were developed in the setting of cancer treatment, three (8%) in the setting of stroke care and two (7%) within rheumatology. Single studies were developed in the setting of cardiovascular disease, dermatology, hip and knee surgery, geriatrics, and spinal cord injury. Sixteen (43%) studies were conducted in a more general setting and did not focus on specific diagnoses (table 1).

### PE structure

As described by others,^11^ structure can be understood as the attributes of a PE setting, such as PE education, resources and the organisation of PE initiatives. PE knowledge, recruitment, resources and physical environment were the most prominent structural attributes reported. Organisation of PE initiatives, including timing and consistency of PE initiatives referred to as the organisational structure, was reported in all studies.

#### PE knowledge

In 23 studies (62%), there was a focus on knowledge and capability on how to engage PE stakeholders.^24–46^ Out of these, a lack of competence was reported as a barrier in nine studies (24%).^26 28 29 31 34 39 40 42 45^ Lack of technical skills,^39^ lack of insight in appropriate PE methods,^26^ lack of understanding legal constraints^34^ and uncertainty on how to incorporate patient experiences,^32^ were also reported as PE barriers. PE knowledge was described as a factor with the potential to work as both a facilitator and a barrier in nine (24%) studies.^24 25 27 32 33 38 41 43 44^ Among the 18 (49%) studies of which lack of PE knowledge was mentioned as a barrier, 8 (22%) reported that multiple stakeholders such as patients, healthcare professionals and/or managers could benefit from increasing their PE knowledge on how to incorporate PE as part of the structure.^27 31 32 34 38 39 44 47^ Similar results were reported in four (11%) studies were PE knowledge was endorsed as a facilitator (table 2).^30 35 37 46^

**Table 2:** Studies highlighting structural attributes as facilitating, hindering or both for PE*†‡

Structural attribute	PE barrier	PE barrier and facilitator	PE facilitator
PE knowledge	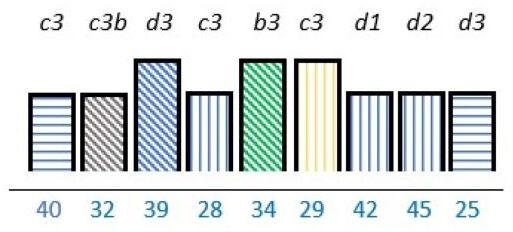	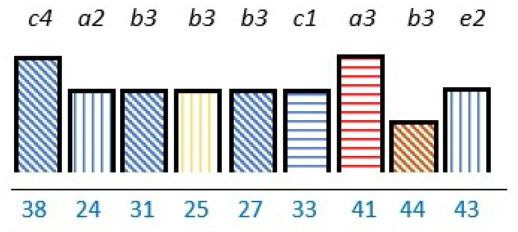	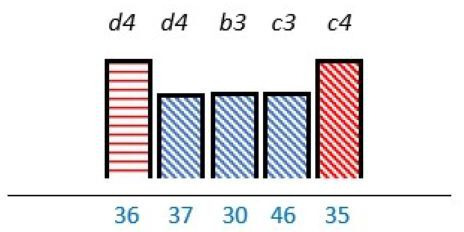
Recruitment and/or representativeness	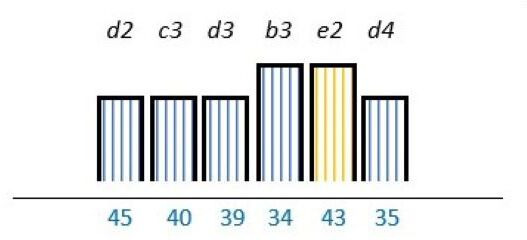	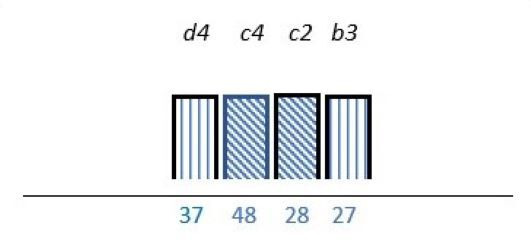	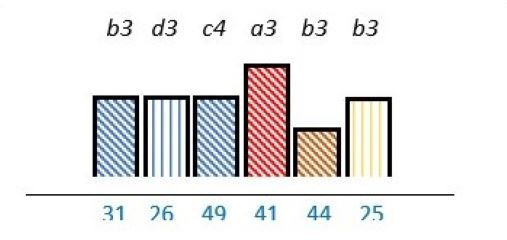
Time and resources	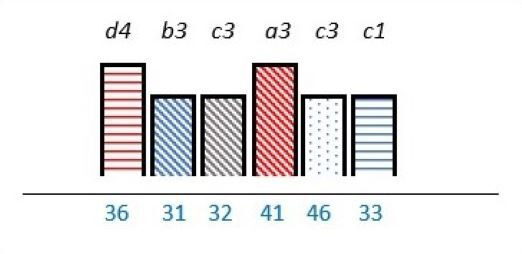	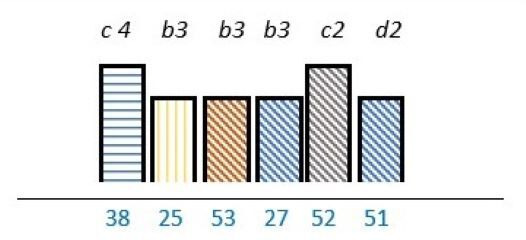	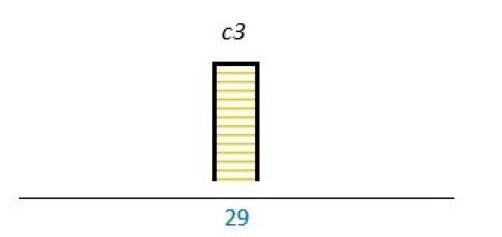
Physical environment	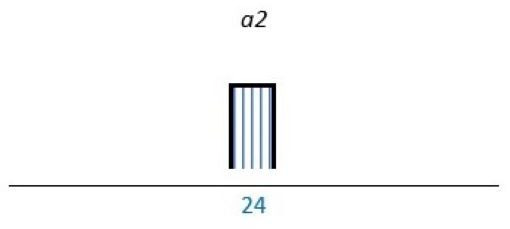	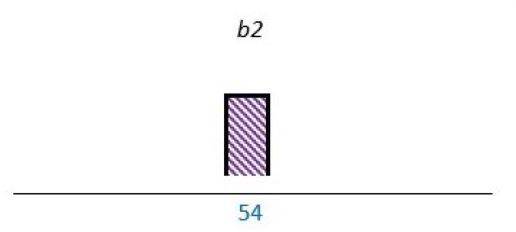	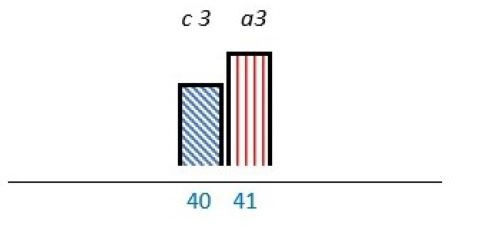
**Ref study**			
Amann *et al* 2018^24^	Armstrong *et al* 2017^31^	Scholtes *et al* 2021^36^	Hwang and Warshaw, 2019^41^
Brouwers *et al* 2017^25^	Armstrong *et al* 2018^32^	Anderson *et al* 2021^37^	McKevitt *et al* 2018^42^
Gagliardi *et al* 2008^26^	Boivin *et al* 2014^52^	Carlsson *et al* 2007^48^	Sharma *et al* 2018^43^
Gurung *et al* 2017^27^	de Souza *et al* 2017^53^	Dayekh *et al* 2022^38^	van der Meide *et al* 2015^44^
Lindblom *et al* 2021^28^	Daouk-Öyry *et al* 2018^54^	Fudge *et al* 2008^39^	Weiste *et al* 2021^45^
Livingston *et al* 2013^29^	O'Donnell *et al* 2019^33^	Galvin *et al* 2020^40^	Whiston *et al* 2019^46^
Neech *et al*, 2018^30^	Greene *et al*, 2018^34^	Hashem *et al*, 2018^49^	
Rise *et al*, 2014^51^	Omeni *et al* 2014^35^		

Affected stakeholder: 
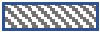
, multiple stakeholders; 
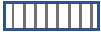
, public or patients; 
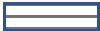
, HP’s managers, leaders; 
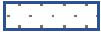
, policy-makers.

Study participants and healthcare setting: (a) HP; (b) PR; (c) HP and PR; (d) HP, managers/leaders, PR. (1) acute care; (2) regional care; (3) specialised care; (4) national/general care.

Study method: 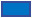 Qualitative; 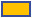, mixed; 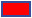, cross-sectional; 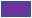, action-research; 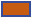, case study; 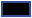, RCT.

*Bar height represents number of participants: Small bar = ≤ n 10, medium bars = > n 10, and highest bars = > n 49.

†Study ref.

‡Blue numbers=study ref.

PE, patient engagement; RCT, randomised controlled trial.

#### Recruitment and representativeness

As shown in table 2, according to 16 (43%) studies, representativeness and/or recruitment had the potential of being both a facilitator and a barrier to meaningful cocreation.^25–28 31 34 36 37 39–41 43–45 48 49^ Of these studies, nine (24%) took place in the setting of specialised care,^25–27 31 34 39–41 44^ three (8%) in regional care^28 43 45^ and four (11%) in the setting of national care.^36 37 48 49^ Insufficient representativeness was reported as only being a PE barrier in six (16%) studies.^34 36 39 40 43 45^ As examples, old age,^37 39^ small numbers of patient representatives with time to spare,^36 39 40^ and professionals recruiting already known patient representatives^45^ were described as a restriction for sufficient representativeness. Sufficient representativeness was highlighted as a facilitator in six (16%) studies,^25 26 31 41 44 49^ and representativeness among multiple PE stakeholders were underlined as a facilitator in four (11%).^31 41 44 49^ Representativeness could be facilitated by recruiting a sufficiently number of engaged stakeholders with the ability to provide general perspectives related to specific PE purposes.^26 27 31 34 37 39 41 43 44 49^ Gender distribution was described in most studies, some of these sought or described a balance of gender.^26 28 35 37 42 44 50^ However, an over-representation of women was described in one study,^44^ and men was described to be more experienced in the role as patient representative in another study.^35^

#### Time and finances

As seen in table 2, time, human resources and/or finances were reported as important structural characteristics in some of the studies. In six (16%) studies, lack of time and/or finances were listed as a barrier.^31 32 36 40 41 46^ For instance, patient representatives could be too overwhelmed by their illness,^41^ or having logistical challenges.^36^ Limited economical resources dedicated to PE from policy-makers was also reported as a PE barrier.^46 51^ Time and/or finances were reported as both a facilitator and a barrier in six (16%) studies.^25 27 38 51–53^ Investing time and resources into training service providers about PE were reported to be facilitators in one (3%) study.^29^ In 7 (19%)^27 31 32 41 51–53^ of the 13 studies in which time and/or finances were addressed, these structural attributes applied to multiple stakeholders.

#### Physical environment

The physical environment was reported as a PE barrier in one (3%) study,^24^ and outlined as a combination of facilitator and barrier to PE in another.^54^ In two (5%) studies, the physical environment was specifically reported to affect patient representativeness,^24 41^ and described in two (5%) to influence multiple stakeholders (table 2).^40 54^ The physical environment, including services and equipment such as transportation and IT systems, was reported as PE facilitators by being innovative in one (3%) study.^41^ In another study, using a room with surroundings where people felt safe and at ease was reported to facilitate PE.^40^

#### Organisational structure

Using combined PE initiatives (eg, patient panel, focus groups and surveys) representing the broader organisational PE activity were frequently reported.^24 25 29 34 37–39 41 42^ Standing committees, boards, councils or panels were often more consistently engaged in PE tasks and integrated as part of the structure than other PE initiatives (online supplemental file 3). However, patient advisory councils were reported in one study to address day-to-day clinical challenges, while governing board patient members tended to handle decisions on a higher operational level.^43^ Of the 17 studies reporting focus groups, workshops and forums, 10 studies associated these initiatives with operationalised PE tasks.^28 29 31 32 34 38 42 50 53^ Consultation meetings were reported as a PE initiative without regular meetings in two studies^33 52^ and action research was used as a framework to organise PE initiatives in three studies (online supplemental file 3).^40 54 55^ Other ways of conducting PE, such as surveys and interviews, were described as more passive forms of engagement,^26 39^ often including undecipherable tasks.

10.1136/bmjoq-2023-002309.supp3 Supplementary data

The PE phase refers to when PE was initiated and the consistency of engagement during a PE process. As seen in online supplemental file 3, patient representatives organised in focus groups, workshops or forums were more likely to participate in an earlier phase of the engagement process than for other PE formats. Early, ongoing PE initiatives were endorsed as preferable over single and passive PE activities,^25 26 36 46 56 57^ with active visible dissemination from PE contributions,^30 32 46 53 58^ and patient representatives taking an active role through the entire PE process.^25 26 31 38 58^

### Process

Process can be understood as activities, tasks, approaches and mechanisms that are performed in a PE structure.^11^

#### PE task

Regarding the studies that comprised a preoperationalised PE task(s), a frequently mentioned task was to share experiences and assist healthcare professionals in the process of prioritising issues of patient concerns.^26 33 37 42 44 49 50 55 59^ It was highlighted that the overall PE purpose was to improve patient experiences of care,^9 28 29 38 40 53 54^ or to contribute to the identification of outcome measures and practice guidelines.^25 31 32 47 60^

#### PE approach

Engagement approaches can be described as heterogeneity of models used in dynamic ways to engage various stakeholders.^11^ Overall, review findings indicate various mechanisms, including interpersonal relationships acting as part of the engagement approach.

Negative interpersonal relationships and experiences were reported as barriers to meaningful PE. Consequently, patient representatives are affected both directly and indirectly in their PE process,^31 32 48^ creating a sense of tokenism and differences in power balance between patients and healthcare professionals.^25 28 30 35 42 44 51 53 55 56^ Examples of barriers to meaningful cocreation were professionals’ taking control over the PE agenda and how patient representatives participated in the engagement process.^39 42 51^ Mutual respect and values, creating a meaningful partnership based on equity among stakeholders, were emphasised as facilitators for PE.^29 31 40 42 44 46 48 50 53^ In some studies, equity was reported in the setting of easily understandable language and respectful communication.^28 33 38 43 53 55^

#### Role clarity

Patient representatives’ desired roles and tasks did not necessarily correspond with the actual role and tasks they ended up performing.^28 40^ Professionals were reported to assume patient representatives to take a consultative role rather than taking part in final decision-making.^26^ A clear description of roles and the importance of accepting different roles and backgrounds was reported as keys to meaningful PE.^25 28 31 33 58^ Training patient representatives in leadership roles,^43^ and in the language and mindset of hospital governance could also facilitate PE.^44^

### PE outcomes

In contrast to hard and operationalised outcomes with clearly defined and measurable effects of PE, the most prominent results of PE were reported as soft outcomes or consequences. Examples of soft outcomes are stakeholder experiences, relationships and capacity building acting as intermediary stages to reach hard outcomes such as improved healthcare quality and cost-effectiveness.^61^

Patient representativeness was described to have an intrinsic value^42^ that may influence decision-making processes in shaping policies, services, guidelines and programmes.^9 32 34 37–39 52 57 60^ Patient representatives can contribute to an increased focus on patient-centred care and argue for other priorities than healthcare professionals traditionally do.^47 53 54 60^ Patient representatives tend to focus more on patient perspectives, such as patient-relevant topics, than healthcare professionals working alone.^47 48 60^ As an example, a patient panel assigned higher importance to avoiding infection than experiencing a disease flare than a physician panel did.^47^ Moreover, patient representatives would focus more on remission than health professionals.^60^ Studies reported that the codesign processes may facilitate mesolevel change by organising varying PE initiatives suitable for certain tasks and process phases.^28 37^ Early involvement of patient representatives was described as facilitating PE and further associated with the greatest impact at the mesolevel.^34 59^

The PE process in itself was reported as a barrier with stigmatising attitudes and power differences as a processual consequence.^26 30 45 49 50 55 56^ Conversely, PE as an adapting process was commented on as important regarding the positive impact on experiences for various PE stakeholders.^24 28 34 36 38 57^ Outcomes such as patient representatives improving their self-esteem and feeling valued when supported by health professionals were emphasised.^29 30 35 42^ Positive experiences resulting from democratic dialogue, mutual respect and equality were reported as outcomes facilitating a meaningful process.^33^ To facilitate a meaningful process, the importance of a common understanding of what PE should contain, clarifying the criteria for success and the timing of involvement were endorsed.^25 50 51 59^ Furthermore, the importance of clarifying types of knowledge contributions expected was embraced.^58^

## Discussion

### Summary of evidence

The objectives of this review were to explore current knowledge about the persons engaged in healthcare cocreation and delivery, investigate facilitators and barriers to PE, and to report PE outcomes. The overall findings suggest that the PE process itself may both promote discrimination and increase stakeholder self-esteem. As supported by others,^62^ structures ensuring sufficient diversity and PE knowledge among all stakeholders, including healthcare professionals, managers and patient representatives, seems to be especially important to facilitate a PE process.

The finding that professional control and prominent use of too-advanced language hinder PE can assist current education and training materials such as The Principles of Community Engagement,^63^ PE Trainings,^64^ ethics frameworks such as PRO-Ethics,^65^ and current PE evaluation tools such as The Public and Patient Evaluation Tool.^66^ The findings may also serve as a basis for the development of new education materials targeting mesolevel PE.

In this review, no studies originated from countries with a low HDI score, implying a possible need for exploring PE processes in developing countries in particular. Both Nepal and India scored ‘medium’ (0.588, 0.630) on the HDI when the studies were conducted and Lebanon had a ‘high’ HDI score (0.747) at the time when the studies were published in 2022^38^ and 2018.^54^ This review may have failed to detect unpublished reports, studies reporting on unfamiliar PE processes, or structures uncommon in western countries possibly detected by a more HDI specific search. Findings suggest that patients and carers from countries with a medium HDI and in a mental health setting describes internalised stigmatising attitudes.^27 56^ Findings described in the study from Nepal imply within-group stigma among patient representatives.^27^ Similar findings are shown in the study from India which reported that PE at microlevel was prioritised over mesolevel by all stakeholders, including policy-makers.^56^ Working towards user centric healthcare services, free from competing interests among stakeholders are described as important first steps to reduce stigmatising attitudes.^27 56^

### Structure

Results indicate that stakeholders’ knowledge regarding the incorporation of PE as part of a healthcare organisation may be an essential structural attribute to facilitate change in practice. This is supported by a recent study on PE within health profession education influencing the microlevel,^67^ where the development and use of context-specific education tools and programmes empowered patients’ to participate in shared decision-making. As part of the organisational structure, the findings further suggest that PE education and training may benefit from focusing on the use of different PE initiatives suitable for specific PE tasks, for instance, by having a standing patient board, which conducts surveys or workshops when needed. In addition to insufficient PE knowledge, uneven power relations are frequently described as hindering PE. Sometimes healthcare professionals and managers perceive PE as a threat.^53^ This threat may serve as a ‘sticky floor’ which holds on to provider-centric structures with unequal stakeholder responsibility and influence. These structures may foster power inequalities and are frequently reported in studies originating from countries with medium, high and very high HDI scores.^30 49 54–56 58^

### Process

In line with a previous study,^68^ included studies indicate that processual experience, such as stakeholder relationships may be difficult to change through education, but may develop as part of the engagement process itself. Studies report that different PE expectations and values among the different stakeholders can serve as an important barrier to PE and that PE stakeholders in some cases tend to place a symbolic value on the decision-making process.^39 42 43 49 50^ In studies were the PE process was described as a barrier, patient representatives tended to be underestimated due to their lack of professional knowledge. It was also reported that the PE process could trigger health difficulties, generate self-stigma and hinder a meaningful cocreation process.^26 30 45 49 50 56^ A recent scoping review concludes that all stakeholders should take on a more progressive role to convert from a pro-forma PE approach to meaningful levels of cocreation.^6^ A meaningful level of engagement embraces empowerment to participate with equity as a core value.^38 44 69^

### Outcome

Even if our findings indicate that patient representatives engaged in the cocreation process may contribute to improved care, there is a lack of research reporting this as hard outcomes. More specifically, a paucity of research report changes in service delivery, improved healthcare quality, cost-effectiveness, health status or overall well-being at the microlevel. On the other hand, soft outcomes such as a change in experiences, stakeholder relationships and stakeholder capacity building were described in the majority of the included studies. These soft outcomes and consequences were similar across PE tasks and organisation of the PE initiatives.

### Strengths and limitations

A strength of our study is the critical appraisal of all included studies, to our knowledge, not applied in scoping reviews before. This may ease the use of the results for future hypothesis generating processes. A limitation is that we only included primary studies published in English, Swedish, Danish or Norwegian. A wide scoping area made the review process long and time consuming. We screened an extensive amount of studies selected from comprehensive searches in the predefined databases, but important studies in other databases may have been missed.

### Future research

Extended hypothesis testing when evaluating rigour in outcome measures is suggested in a previous study.^70^ The findings from this review may work as a fundament when considering additional variables to test. Future research is proposed to explore how consistent and diverse PE initiatives engaging multiple stakeholders may promote respect and equity among PE stakeholders. An important next step could also be to explore HDI scores related to PE by including more specified searches for this purpose. In addition, mesolevel PE may benefit from research focusing on structured PE education and training with possible outcomes at different levels of care and how these outcomes may be experienced by stakeholders. Studies have reported diversity among stakeholders as a facilitator of PE, but few have reported on gender differences among stakeholders in general, or patient representatives in particular.

## Conclusions

This scoping review demonstrates a lack of research describing clearly defined outcomes identifiable for patient representatives, healthcare professionals, managers and patients. The PE process may flourish further through education, training, experience and stakeholder diversity. Sufficient stakeholder representativeness and PE knowledge are reported as the most noteworthy structural attributes to facilitate equity and a meaningful cocreation process. Overall, earmarked finances ensuring sufficient PE representativeness and knowledge among all stakeholders is a cornerstone of integrating PE as a part of a healthcare structure in any setting.

## Data Availability

No data are available.
