# Supplementary material for: Patient engagement in the development and delivery of healthcare services: a systematic scoping review
Source: BMJ Open Qual. 2023 Jun 27;12(2):e002309. doi: 10.1136/bmjoq-2023-002309 (PMC10577732; doi:10.1136/bmjoq-2023-002309)
Supplement: Supplementary data [file bmjoq-2023-002309supp001.pdf]

Supplemental file 1 Search-strategy

Medline

Database: Ovid MEDLINE(R) and Epub Ahead of Print, In-Process, In-Data-Review & Other Non-Indexed Citations and Daily <1946 to October 03, 2022>

Search Strategy:

----

1 exp Patient Participation/ (28922)

2 exp Health Planning/ (369941)

3 exp Health Policy/ (113709)

4 exp "Delivery of Health Care"/ (1198519)

5 exp Organizational Policy/ (14505)

6 exp Health Services/ (2369258)

7 exp Decision Making, Organizational/ (11237)

8 exp Decision Making/ (227165)

9 user engagement.mp. (840)

10 involvement.mp. (511585)

11 representatives.mp. (30091)

12 committees.mp. (36899)

13 Quality.mp. (1461621)

14 delivery.mp. (695739)

15 advisory councils.mp. (119)

16 2 or 3 or 4 or 5 or 6 or 7 or 8 or 9 or 10 or 11 or 12 or 13 or 14 or 15 (5361480)

17 1 and 16 (28922)

18 limit 17 to yr="2005 -Current" (19302)

19 limit 18 to (danish or english or norwegian or swedish) (18205)

20 limit 19 to ("young adult (19 to 24 years)" or "adult (19 to 44 years)" or "young adult and adult (19-24 and 19-44)" or "middle age (45 to 64 years)" or "middle aged (45 plus years)" or "all aged (65 and over)" or "aged (80 and over)") (8127)

21 exp Primary Health Care/ (185856)

22 exp General Practitioners/ (10012)

23 20 not (21 or 22) (7021)

24 limit 23 to dt=20200601-20221004 (559)
