# Supplementary material for: Patient engagement in the development and delivery of healthcare services: a systematic scoping review
Source: BMJ Open Qual. 2023 Jun 27;12(2):e002309. doi: 10.1136/bmjoq-2023-002309 (PMC10577732; doi:10.1136/bmjoq-2023-002309)
Supplement: Supplementary data [file bmjoq-2023-002309supp002.pdf]

Supplemental file 2 Quality appraisal of the included studies[a,b]

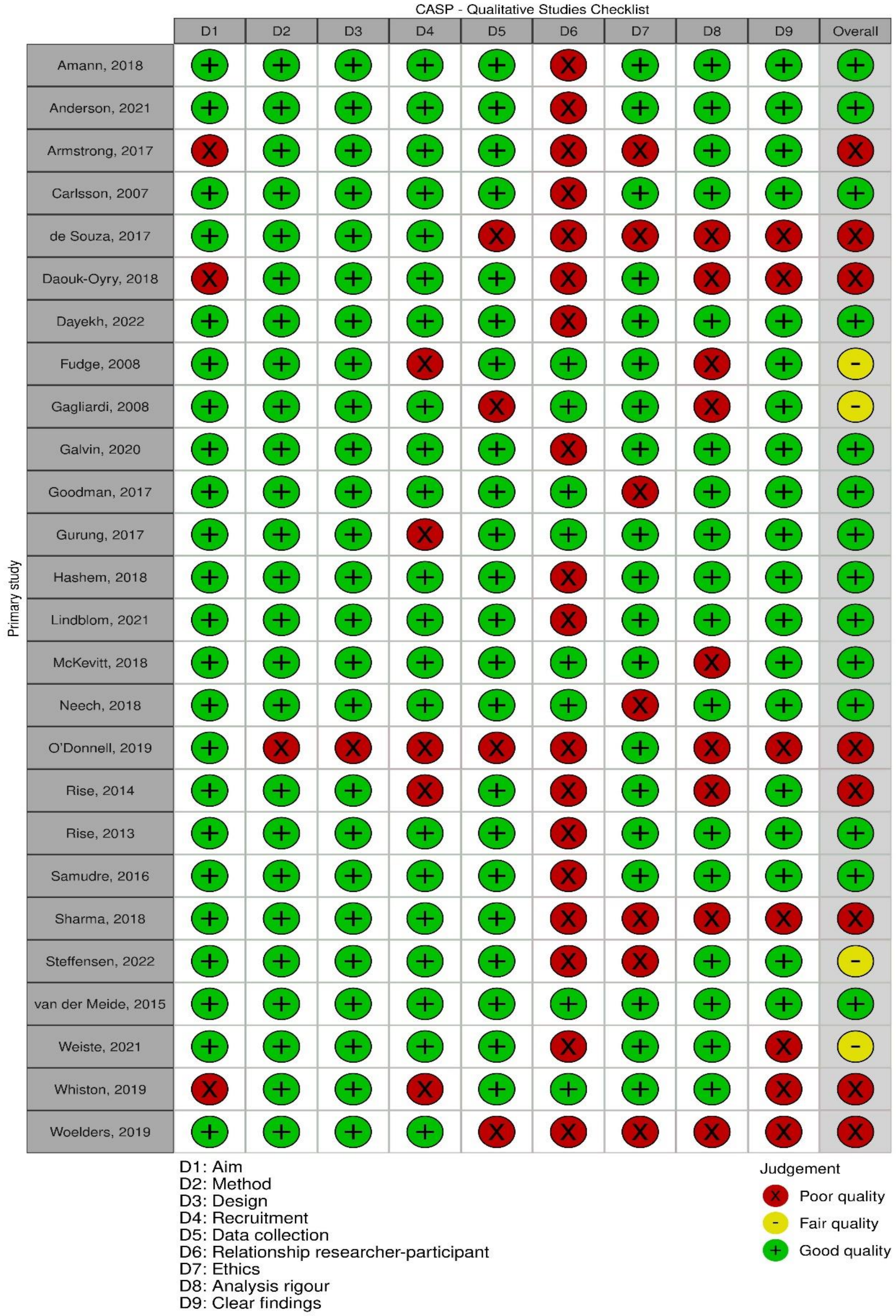

[a]The response categories C= Can't tell and N=No were collapsed to no, < two "no" = good quality, < three "no" = fair quality and ≤ three "no" = poor quality. [b]Robvis[23] was used to create visual quality assessment tables.

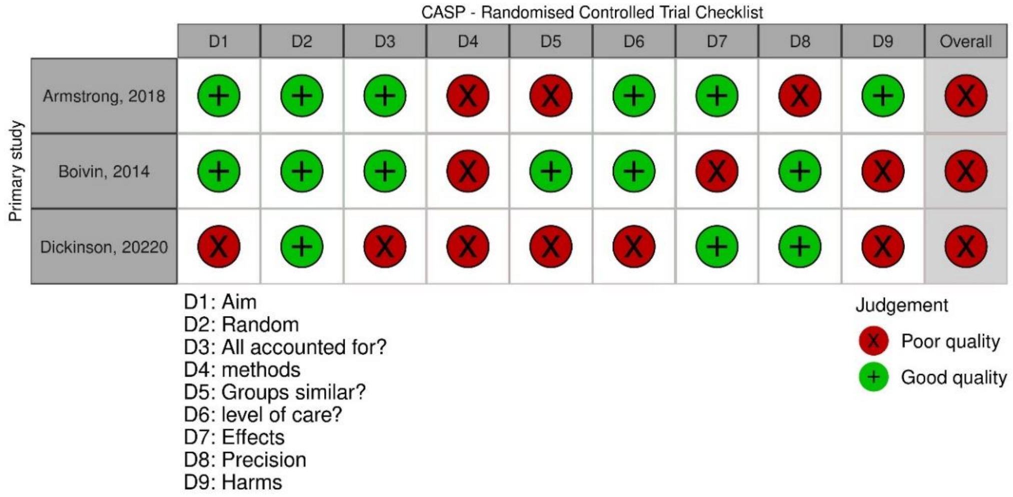

[a]The response categories C= Can't tell and N=No were collapsed to no, < two "no" = good quality, < three "no" = fair quality and ≤ three "no" = poor quality. [b]Robvis[23] was used to create visual quality assessment tables.

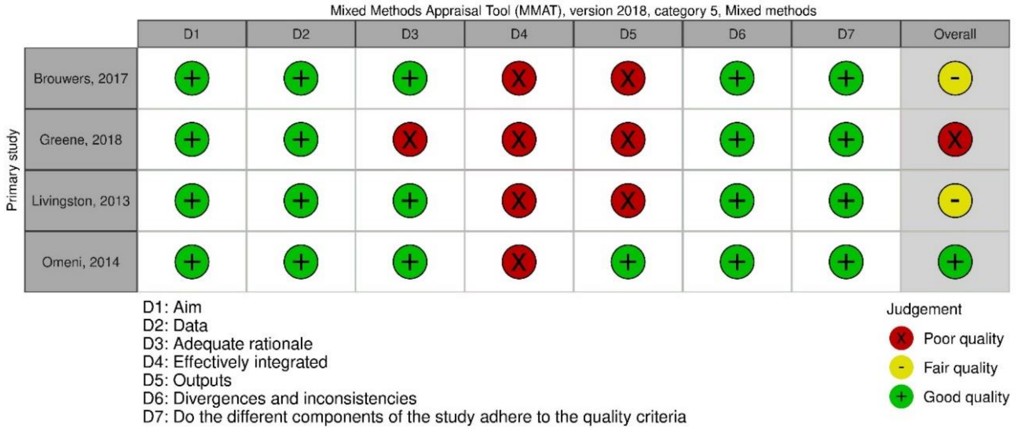

[a]The response categories C= Can't tell and N=No were collapsed to no, < two "no" = good quality, < three "no" = fair quality and ≤ three "no" = poor quality. [b]Robvis[23] was used to create visual quality assessment tables.

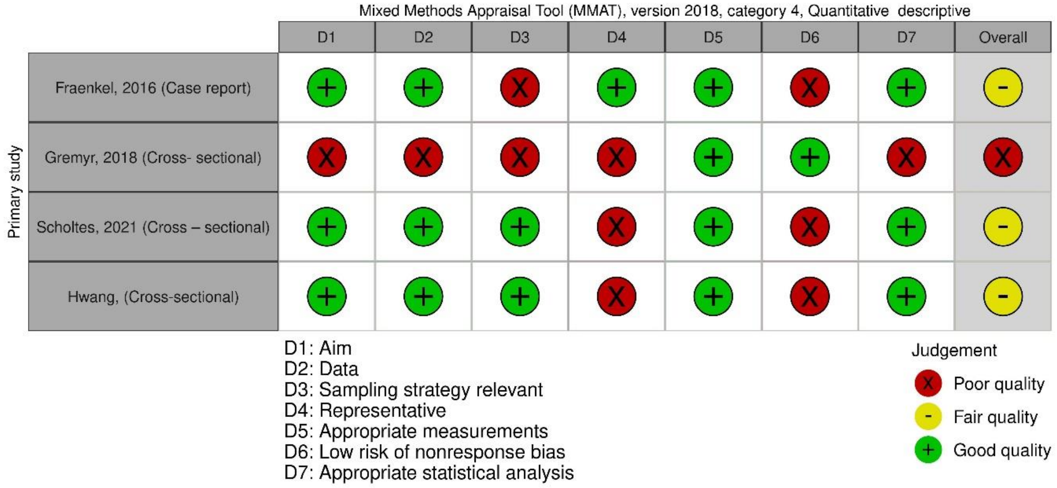

[a]The response categories C= Can't tell and N=No were collapsed to no, < two "no" = good quality, < three "no" = fair quality and ≤ three "no" = poor quality. [b]Robvis[23] was used to create visual quality assessment tables.
