# Supplementary material for: Patient engagement in the development and delivery of healthcare services: a systematic scoping review
Source: BMJ Open Qual. 2023 Jun 27;12(2):e002309. doi: 10.1136/bmjoq-2023-002309 (PMC10577732; doi:10.1136/bmjoq-2023-002309)
Supplement: Supplementary data [file bmjoq-2023-002309supp003.pdf]

Supplemental file 3 Organisation of PE initiatives related to consistency and timing of PE tasks

**Organisational structure**			**PE[a] phase**						
	**PE task (ref) n**		**PE timing (ref) n**				**Consistency of PE initiatives (ref) n**		**Total[b] n= 49 (N =148)**
Organisation of PE initiatives	*Clearly defined/ operationalised*	*Undecipherable*	*Planning*	*Delivery*	*Evaluation / feedback*	*Unspecified*	*Consistent PE initiative*	*Inconsistent PE initiative*	
Focus groups/workshops/ forum	[25, 28, 29, 31, 32, 53, 34, 38, 43, 50] n=10	[24, 30, 56, 48, 39, 41, 45] n= 7	[25, 28, 30, 31, 32, 53, 34, 38, 50] n=9		[24, 28, 29] n=3	[56, 48, 39, 41, 42, 45] n=6	[29, 30, 48, 38, 41, 42, 50] n= 7	[24, 25, 28, 56, 31, 32, 53, 34, 39, 45][c] n=10	**18 (52)**
Committee/board/panel/ council	[29, 60, 47, 57, 37, 38, 49, 42, 43, 58, 44] n=11	[51, 36, 39, 41] n=4	[60, 47, 37, 38, 49, 43] n=6	[37] n=1	[29, 44] n=2	[51, 36, 39, 41, 42, 58] n=6	[29, 51, 47, 36, 37, 38, 49, 41, 42, 43, 58, 44] n=12	[60, 57, 39] n=3	15 (45)
Consultation/discussion meeting.	[33] n=1	[52] n=1	[52, 33] n=2	[33] n=1	[52] n=1			[52, 33] n=2	2 (8)
Survey/ interview	[25, 26, 34, 59, 37, 38, 42] n=7	[24, 35, 39] n=3	[25, 34, 37, 38] n=4		[24, 26, 59, 35, 37] n=5	[39, 42] n=2	[42] n=1	[24, 25, 26, 34, 59, 35, 37, 38, 39] n=9	10 (31)
Action research, Experienced-based co-design, Mystery patient	[54, 40, 55] n= 3		[54, 40] n= 2		[55] n=1			[54, 40, 55] n=3	3 (9)
Delphi Technique Experiment	[46] n=1		[46] n=1					[46] n=1	1 (3)
**Total n = 114 (N=148)**	**25 (33)**	11 (15)	**19 (24)**	2 (2)	10 (12)	9 (14)	15 (20)	**23 (28)**	

[a]PE: Patient engagement.[b]n = primary studies, N = occurrences. [c]Study [24, 25, 29, 34, 37, 38, 39, 41, 42] reported diverse PE initiatives and are reported in more than one PE initiative. Lighter colours indicate fewer studies, and darker indicate more studies.
